# Assessing the Belt and Road Initiative as a narrative: Implications for institutional change and international firm strategy

**DOI:** 10.1007/s10490-021-09757-x

**Published:** 2021-03-07

**Authors:** Tomas Casas-Klett, Jiatao Li

**Affiliations:** 1 University of St. Gallen, St. Gallen, Switzerland; 2 Department of Management, Hong Kong University of Science and Technology, Clear Water Bay, Hong Kong

**Keywords:** Belt and Road Initiative, Globalization, Narrative economics, Institutions, China

## Abstract

This article explores the role of narratives as drivers that guide the institutional change associated with globalization and deglobalization. For China’s Belt and Road Initiative (BRI) to succeed as a driver of institutional change in favor of globalization, it must pass the narrative “virality” test and successfully contend with competing narratives. Rival narratives will be launched by firms and organizations worldwide that expect to win or lose from deglobalization or from new forms of globalization. This study develops a useful framework for establishing the extent to which China’s BRI is a genuine narrative or just a story. In this regard, four testable propositions are put forth to ascertain whether the BRI is values-based, extends an invitation to participate, is open-ended, and is associated with economic performance for both Chinese and non-Chinese participants. The analysis of the BRI-related institutional change that leads to globalization applies a theoretical lens centered on the narrative economics perspective and on the institution-based view and political economy perspective. Implications for BRI stakeholders, international business practitioners, and international business scholarship are outlined.

## Introduction

This article addresses two research questions. First, how are the institutional changes associated with globalization and deglobalization influenced by the contexts of narratives in general and the Belt and Road Initiative (BRI) in particular? Second, what factors drive the virality of the BRI as a narrative and the impact on international firm strategy? These questions are relevant for scholarship and policymaking and have important practical implications for the conduct of international business and for firms’ strategies.

Researchers and practitioners alike have wondered exactly how the present economic, geopolitical, political, and technological disruptions will reshape institutional arrangements. Executives, thought leaders, and policy-makers in China, Russia, Switzerland, and the United States, with whom we have engaged in discussions, view 2020 as an inflection point for globalization. They note that many outcomes are still possible, including both greater integration and greater fragmentation. For instance, China could decide to deemphasize the BRI and focus its resources inward on domestic recovery. Alternatively, China could seek ambitious institutional change with European partners through the EU-China Comprehensive Agreement on Investment (CAI) finalized in December of 2020 or in digital cooperation (European Commission 2020), and with Asian partners of the recently signed 15-nation Regional Comprehensive Economic Partnership (RCEP), hailed as the “world’s largest free trade bloc” (State Council 2020).

This study attempts to explain deglobalization and globalization (jointly referred to as de/globalization) in relation to the BRI. It employs the theoretical lens of narrative economics, along with an institution-based view and, to a lesser extent, a political economy perspective to address conceptual problems associated with social, political, geopolitical, economic, and other aggregate forces that disrupt international business practices and force the reevaluation of generally accepted approaches.

Narratives are coherent desciptions with structures built arround sets of circumstances, actions and actors: moreover, they point to outcomes and can be employed “to predict the new state of the world.” (Abell 2004: 295). To Shiller narratives are an “easily expressed explanation of events” and are “exogenous in economics” (2017: 968). Specifically, narratives are drivers and causes (of economic fluctuations or institutional change). They derive their power from the ability to engage the human mind and heart and therefore affect behavior and decision-making. We suggest that narratives—as drivers of institutional change—enable a deeper understanding of the current de/globalization context and of the BRI’s prospects.

## Drivers of de/globalization and narratives

### Drivers of globalization and deglobalization

Scholarly work has identified multiple drivers or antecedents of de/globalization and its institutional changes. We conceive of these drivers as either narratives, such as nationalism, that develop into institutional forms (e.g., trade barriers) or as exogenous forces that narrativize (e.g., e-commerce and search engine technologies and business models become data or national security narratives). Examining how exogenous drivers are cast as narratives along the globalization to deglobalization spectrum is helpful.

The contextual drivers of deglobalization are usually thought of as being economic, political, and sociocultural (Meyer 2017). They are treated as the result of “a specific conjunction of political-economic conditions” (Kobrin 2017: 160) that can be explained by political science theories, such as realism (Witt 2019). The key point is that facts and forces have a particular economic impact when articulated as narratives because narratives can more easily become viral and develop claims on individuals’ cognition and affect (Casas and Buckup 2018; Shiller 2017). Narratives influence rules and institutions, in turn yielding winners and losers in business. “Power shifts towards those who understand and deploy narrative,” and that applies to both governments and multinationals (Allenby and Garreau 2017) because narratives have explanatory power, as Shiller (2017, 2019) has demonstrated. According to Abell (2004: 287), narrative explanations can be taken as “an alternative to…better established variable-centered explanations.” We argue that narratives matter in international business because they can define the critical context.

A narrative’s plausibility can change in accordance with the changing attitudes toward de/globalization (Jones 2004; Kobrin 2017; Meyer 2017). Table 1 shows some of the main factors—both narratives and narrativized exogenous forces—that drive globalization and deglobalization. As their plausibility waxes and wanes, so moves the de/globalization pendulum. The present swing seems to be toward deglobalization despite the net loss in general welfare and economic activity that it produces in aggregate. For instance, politics (Witt 2019) and geopolitical considerations (Allison 2017) were among the drivers that caused the U.S. government to announce in March 2018 a high-impact deglobalization measure: a 25% tariff on imports of steel products and a 10% tariff on imports of aluminum products (Salotti et al. 2019). Although exemptions and amendments were subsequently implemented, the first-order effect was to anoint U.S. steel and aluminum producers as significant winners and exporters from the EU, China, and Russia as significant losers. Once enacted, rules and institutions tend to persist because of the power of narratives as much as because of “the distributive benefits that they provide to the underlying social or political coalitions” (Hall and Thelen 2009: 7).

**Table 1:** Drivers of globalization and deglobalization

**Globalization**	**Deglobalization**
**Institutional drivers:**	
- Supranational institutions in the postwar period, i.e., World Bank, IMF, GATT, WTO since 1994 (Cuervo-Cazurrra et al. 2017; James 2018; Kobrin 2017) - Agreements among public regulatory authorities and private standard setters, e.g., International Organization for Standardisation (ISO) (James 2018) - Washington Consensus (Cuervo-Cazurrra et al. 2017; Kobrin 2017) - Born globals, entrepreneurs enabled by emerging global networks (Prashantham et al. 2017) - The Belt and Road Initiative (BRI)	- Renewed emphasis on national sovereignty; trade and investments force nations to adjust regulatory regimes (Meyer 2017) - Protectionism and discrimination against foreign commerce (Braga 2018) - Protectionist trade tariffs, railroad tariffs, hygiene requirements for imports, quotas (James 2018) result in world trade collapse (van Bergeijk 2018) - Erosion of dominant currency arrangements (van Bergeijk 2018)
**Domestic political drivers:**	
- Important political changes and reforms in the 1980s and 1990s to a neo-liberal agenda and reforms, such as those implemented by Thatcher (Cuervo-Cazurrra et al. 2017; Kobrin 2017) - Economic growth and improved human rights (Potrafke 2015)	- Democratic calls for deglobalization (van Bergeijk 2018) and a surge in support for protectionist and nationalist political parties and leaders (Meyer 2017) - A loss of manufacturing jobs and a shrinking middle class as motivation for trade barriers (Kobrin 2017) - Reaction to the economic dislocations resulting from globalization (Kobrin 2017; Stiglitz 2005) - Resistance to the power of MNEs relative to that of national governments and other stakeholders (Meyer 2017; Vahlne et al. 2018) - Consumer health and safety, environmental, and social responsibility standards invoked as a motivation for trade barriers (Meyer 2017)
**Geopolitical drivers:**	
- The end of the Cold War and the integration of the Eastern bloc into the world market (Cuervo-Cazurrra et al. 2017; Kobrin 2017) - China’s integration into the world market - Regional integration (e.g., Maastricht treaty and NAFTA) (Cuervo-Cazurrra et al. 2017; Kobrin 2017)	- Geopolitical rivalry (China-U.S., sanctions, etc.), the Thucydides trap (Allison 2017) - National security concerns invoked as a motivation for trade barriers (Meyer 2017) - Erosion of America’s hegemony (van Bergeijk 2018)
**Technological drivers:**	
- Lower communication costs and general spread of technology (Bang and Markeset 2012) - Networks as a distinct mode of economic organization making the closure of national borders costly and ineffective (Kobrin 2017) - Digital e-commerce, social networks, and entertainment (Braga 2018: 2) - Supply chain expansion via reduced transportation costs (Braga 2018; Kobrin 2017; van Bergeijk 2018)	- Technology available to support reshoring (Braga 2018; Kobrin 2017; van Bergeijk 2018) - Rules on local data storage and processing and cross-border transfers (Braga 2018)

As a general principle, the narrative attached to an institutional, political, geopolitical, or technological force is a key determinant of whether such a force drives further institutional change. Many of the forces and facts described in Table 1 can turn into drivers of either globalization or deglobalization. For instance, technologies such as 3D printing or robotics might favor globally optimized supply chains or the opposite—reshoring. However, the influence of many drivers is unambiguous. For example, Table 1 identifies supranational institutions, such as the World Bank or the WTO, as drivers (Cuervo-Cazurrra et al. 2017; James 2018; Kobrin 2017), and they are clearly committed to globalization. Lower communication and transportation costs and the spread of technology, especially technology associated with digital flows (Bang and Markeset 2012; Braga 2018) and born-global entrepreneurs (Prashantham et al. 2017), are all forces that will likewise naturally turn into narrative drivers of globalization. In contrast, deglobalization’s drivers include national security concerns and geopolitics (Allison 2017; Meyer 2017; van Bergeijk 2018) or domestic politics that seek to protect people and firms losing from globalization (James 2018; Kobrin 2017; Stiglitz 2005).

When the narrative associated with a set of drivers goes viral and manages to win in the market for narratives, institutional change and even institutional reversal can occur fairly quickly, as shown by the rapid resurgence of the deglobalization sentiment. In this vein, Braga (2018) asks whether peak globalization was reached in 2007. However, the cyclical conceptualization of alternating globalization and deglobalization (Jones 2004; Kobrin 2017; Meyer 2017) expects a return of globalization. The only questions are when and how. Can the BRI emerge as a force—as a narrative driver for institutional change that helps swing the pendulum toward globalization? This question has no clear answer. For instance, the extent to which a BRI-funded railroad in Southeast Asia delivers a positive return matters, but the winning narrative might still be one emphasizing environmental damage or corruption. The realization of the aims of the BRI’s globalization and institutional change will depend at least in part on the narrative that will emerge to describe it and its success in attracting sustained attention.

### Narratives on institutional change

Institutions are “humanly devised constraints that structure political, economic and social interaction” (North 1991: 97). Peng (2002) demonstrates that an institution-based view explains differences in business strategy across countries and regions. Since 1949, Chinese institutions have been viewed as outliers relative to institutions of other developing economies, even within the socialist camp (Naughton 2007: 9). More recently, they are still conceived of as distinctive (Redding and Witt 2007; Witt 2010), with a “unique blend of central orchestration and local devolution” (McNally 2006; cited in Peck and Zhang 2013: 360). This description includes all-important informal institutions, such as social networks, that might persist even after formal institutions mature; therefore, institutions in China and East Asia are different from those in the West (Li and Xie 2019). A question about the BRI, or “China’s variety of internationalization,” is whether it represents a unique example of institutional globalization. Institutional change in China is mostly designed on the country’s terms, such as when the China International Development Cooperation Agency (CIDCA) is involved, but is also open to multilateral influence, such as the Asian Infrastructure Investment Bank (AIIB). Hall and Thelen (2009: 9) emphasize that a “sophisticated understanding of institutional change” is “not incompatible with dynamic views of the political economy.” To understand the unfolding of the BRI and the nature of China’s globalization institutions, considering the theoretical link between the institutional and political economy perspectives is helpful.

Whereas states appear to set “the rules of the game” (Wilkins 2010: 640), institutional processes can alternatively be conceived of as outcomes of initiatives driven by firms and other economic agents seeking to improve their positions. Institution-backed globalization initiatives often yield very specific winners and losers (Hillebrand 2010), and the BRI promises to be no exception. The varieties-of-capitalism literature recognizes both the relationships among institutions, politics, and the economy and the impact of those relationships on economic outcomes (Hall 2015: 1). The BRI institutions, and institutions everywhere such as the interagency Committee on Foreign Investment in the United States (CFIUS), bilateral free trade agreements (FTAs), or economic sanctions regimes, can be viewed as international institutional manifestations of domestic concerns. They are the outcomes of domestic political economy maneuvering and, once the rules of globalization have changed, simultaneously represent essential contexts, constraints, and opportunities for international firms. Institutions condition the formulation of international strategy because institutions can be shaped (e.g., tariffs or FTAs can be lobbied for) and require strategic adaptation (i.e., how to overcome the effects of tariffs or benefit from FTAs).

Institutional arrangements favoring globalization (e.g., the BRI or cross-border e-commerce) or deglobalization (e.g., tariffs or subsidies to domestic champions) reflect the interests of those who have prevailed in domestic policy disputes. Wilkins (2010: 640) details certain types of rules that determine market incentives: regulations protecting national industry sectors, antitrust restrictions, labor regulations, financial sector and competition strictures, and corporate governance requirements. Many internationally active firms maintain government affairs departments that lobby politicians in the hope of shaping the rules of global institutions to their advantage. The World Intellectual Property Organization’s (WIPO) special legal protections for databases and the elimination of developing country rates at the Universal Postal Union (UPU) are prime examples.

## The BRI as a globalization narrative

### Is the BRI a narrative?

Narratives, as understood from the literature reviewed in this article (e.g., Shiller 2017, 2019; Hagel 2011; Denning 2006; Fisher 1984), are open ended and incessantly unfolding accounts that invite participation and action: when narratives successfully go viral, they impact both behavior and economic outcomes. An increasingly critical aspect of a firm’s strategy is to conceive of or capture, and then groom and nurture, narratives consistent with its business objectives. In the context of international business, narratives will be developed and deployed to shape the institutional changes of de/globalization and support firm performance. Firms might seek tariffs (e.g., in the U.S. steel production sector), open markets (e.g., in the U.S. technology sector), or BRI participation (e.g., in Switzerland’s financial services industry). The different globalization narratives will play out within their domestic political settings with the aim of encouraging the desired institutional change.

Narratives that achieve virality can acquire a life of their own with intended and unintended outcomes. However, narratives without a call to participate make weaker claims on their recipients’ cognition and affect, reducing the chance that the narratives shape both domestic and international institutional change. The possibility of participation brings a commitment to institutional change but is accompanied by the risk of unintended interpretations that can divert the narrative’s effects. Take, for example, a narrative intending to position the BRI as an inclusive global public good (Han and Casas 2018). Researchers may find evidence that supports this argument, such as positive formal institutional effects on export performance to BRI countries by minority-owned SMEs in China’s Western provinces (Li et al. 2019). At the same time, entities who are not in a position to participate or are excluded from the shaping of the BRI, or those who for whatever reason have been disappointed, may suggest counternarratives about “China first” or “one-way traffic” (Wuttke 2017).

Narratives and their virality do not emerge out of thin air. They are strategically nurtured, often by firms and vested interests seeking to “set changes and transformations in motion that have impact on the big picture” (Boje 2008: 13). The BRI started in the context of regional development policies in China and from debates in the Chinese media and among academics (Summers 2016). For ideas to play a transformative role in society, it is very helpful if they can be packaged as a narrative with virality potential. Virality factors include being values-based (Denning 2006; Fisher 1984), extending an invitation to participate and being open-ended (Hagel 2011), and being associated with economic performance (Shiller 2017). Does the BRI fit the bill?.

The narrative perspective can address “big questions” in international businesses, such as what determines firms’ international success or failure (Peng 2004: 99). Research on narratives can be realist (narratives are used to understand phenomena that exist independently of the narratives per se) or interpretative (social construction in and through narratives), as Vaara, Sonenshein, and Boje (2016: 500–501) explain. A realist narrative example at the firm level would be “the BRI helps explain the acceleration of certain Chinese (and foreign) firms’ internationalization strategies.” An example of an interpretative narrative, at the contextual level, would be “China’s BRI is shaping globalization in the 21^st^ century.” If the BRI becomes a viral narrative, it will have implications for firms’ international strategy choices. Chinese and international organizations will interpret BRI in light of their own narratives which they will then align with the narrative of the BRI and thus contribute to its development. Table 2 considers the four virality factors previously reviewed and assesses the BRI’s potential as a narrative in contrast to its potential as a story.

**Table 2:** Virality factors: Is BRI a story or a narrative?

**Virality factors**	**References**	**BRI as a story**	**BRI as a narrative**
**1. Values-based**	Narratives “transmit values” (Denning 2006: 46), have meaning (Fisher 1984)	BRI values are neither clear nor universal; a European ambassador in Moscow notes that “[the] BRI reflects Chinese values, not universal values”^a^	The BRI’s values are meaningful and shared
**2. Invitation to participate**	“(N)arratives spark action” (Denning 2006: 44) and invite participation (Hagel 2011)	The BRI is of interest, and opportunistic deal-making is possible; bilateral BRI MOUs describe what to expect	The BRI informs strategy at firms engaged in international business and seeks diverse contributions and Chinese and non-Chinese leadership alike
**3. Open-ended**	Future scenarios and long-term implications (Denning 2006: 47); “consequent state of affairs” (Czarniawska 1998: 2); the continually unfolding narrative is determined by our choices (Hagel 2011)	The BRI is about connectivity and specific business opportunities and deals	The BRI is evolutionary, e.g., the 2017 Belt and Road Forum focused on connectivity, whereas that in 2019 focused on institution-building
**4. Economic performance**	Strong claims on cognitive and affective bandwidth create virality and impact business behavior and strategy (Casas and Buckup 2018; Shiller 2017)	Project-based, gainful deals for Chinese and, at times, non-Chinese political or economic coalitions	The new institutions of the BRI provide gains for Chinese and foreign business interests who then associate themselves with the BRI narrative

^a^Private conversation with European Ambassador in Moscow in April 2018

Then, a matter of relevance is that the BRI’s narrative is consistent with globalization. Moreover, China puts forth its contesting narratives from a position of strength, given that China today depends on the world less than the world depends on China. After 40 years of economic reform and rapid development, China has emerged as one of the largest economies in the world, a center of technology innovations and global value chains, and an attractive domestic market with hundreds of millions of middle to high income consumers attracting the attention of foreign investors. This reversal is surprising given China’s strong dependencies on other countries until not long ago (Woetzel et al. 2019: 5) and quite a visible change during the recent pandemic. At the same time, the Trump administration made a U-turn from America’s own liberal narrative endorsing free trade. Whether the Biden administration will pivot to more conventional positions remains to be seen.

In any event, the fate of the BRI’s globalizing narrative will depend on the extent to which Chinese and foreign firms and their coalitions perceive the BRI as being a narrative and in their self-interest. After all, this narrative will contend with counternarratives. For the BRI to be confirmed as a globalization narrative (independent variable) driving institutional change (dependent variable), it must have the ability to go viral. Four testable propositions are suggested in this regard and are summarized in Table 2.

### How BRI narratives go viral and drive institutional change

Ahlstrom (2012) suggests that narratives and interpretive perspectives that structure observations can contribute to management theory. Of special interest for de/globalization is the promise that narrative analysis sheds light on organizational stability and change (Vaara et al. 2016: 495). Shiller’s (2017, 2019) work on “narrative economics” focuses on the concept’s development and virality. He also suggests that, although “narrative” and “story” are often understood as synonyms for “a sequence of events,” narratives are the “*telling* of a story” (Shiller 2019: 3). Narratives are also much more than that because they have “meaning for those who live, create, or interpret them” (Fisher 1984: 2).

#### Values-based

Interpretation occurs through the filter of values. Hence, the first factor in a narrative’s virality is its suitability as a device for transmitting values (Denning 2006: 46). This factor distinguishes narratives from stories—cognitively and affectively, narratives run much deeper than stories. The institutions of the liberal order that America constructed after World War II exemplified a unique set of Western values. The BRI values will be different but must be articulated and clear and must transcend China and mere economic performance (as described in Proposition 4). Specific values embodied in the BRI as a globalization narrative could include a commitment to the environment and sustainability, two-way trade, or digital inclusiveness. Presently, the BRI-promoted values of “community,” “common destiny,” and “shared interests” still ring hollow to many (Hillman 2018). Values must have substance and consistency. For instance, whether projects are implemented with partners from Switzerland or Bosnia-Herzegovina, they must consistently reflect the BRI narrative core values.

##### Proposition 1:

For the *BRI narrative of globalization to go viral*, it must live out a consistent set of values that is meaningful and legitimate for Chinese and host country stakeholders, including business executives, government officials, and citizens at large.

#### Invitation to participate

Action has been conceptualized in terms of three elements: an original state of affairs is followed by an action that leads to a new state (Czarniawska 1998: 2). For organizations, the ability to influence a new state (of consumer behavior or institutional change) will obviously spark action. For the BRI to be a narrative, it must ignite actions by key stakeholders and “reach out to embrace as many participants as possible” (Hagel 2011). By the end of January 2020, the China Belt and Road Network (2020) reported 200 cooperation documents signed with 138 countries and 31 international organizations. BRI participants include national governments, international institutions, firms of the signatory countries, and, increasingly, parties from third countries, including multinational enterprises (MNE) (Zhang 2019). That the BRI narrative of globalization translates into increasingly participatory institutional arrangements is, for instance, evidenced by the National Development and Reform Commission (NDRC) policy of “third-party market cooperation” approved in 2019; this policy invites foreign lenders to participate and shape the governance of BRI projects (Dann et al. 2019). Because China will be the most powerful party in any bilateral relationship, calls for multinational participation send the signal that the BRI is a narrative “for” and “by” Chinese and non-Chinese stakeholders alike.

##### Proposition 2:

For *the BRI narrative of globalization to go viral*, it must be a call to action that invites both Chinese and non-Chinese participation.

#### Open-ended

Unlike stories that move toward a foregone conclusion, narratives are open-ended (Hagel 2011). The manner in which narratives unfold depends on the actions, participation, and choices of their target audience as much as on the strategies of the organizations that initially conceived of or promoted the narrative. Linking back to Proposition 1, China might have a values advantage if the BRI is flexible, adaptable, and experimental. An open-ended narrative of globalization means that the BRI narrative evolves and institutions quickly adjust to disruptions, such as the pandemic, with a new focus on public health (Wu and Wong 2020). It also means the agility to decisively incorporate emergent technologies, such as e-commerce or digital currency (such as the e-RMB), as signaled in the 2019 forum *Co-building twenty-first Century Digital Silk Road* (DSR) hosted by the Cyberspace Administration of China (CAC) and the NDRC. Countries that have signed DSR-specific MOUs include Cuba, Saudi Arabia, and the United Kingdom (Eurasia Group 2020). A BRI narrative that evolves is not fixed by design, and an adaptive set of matching institutions will offer complementary new modes of globalization.

##### Proposition 3:

For *the BRI narrative of globalization to go viral*, it must be open-ended and evolutionary, leading to rules that effectively address disruptions and emergent phenomena, such as new technologies.

#### Economic performance

The success of the BRI’s narrative will be determined to a large extent by whether its globalization institutions actually enhance firms’ business performance and general human development in BRI countries, not just in China. This point is presently the subject of significant contention. For instance, the Center for Strategic and International Studies (CSIS) Reconnecting Asia Project notes that, although the BRI claims to endorse universal values, only approximately 11% of Chinese-funded transportation projects tracked have been won by non-Chinese companies (Hillman 2018). Is that performance sufficient to support interest in the narrative? In boardrooms the world over, business leaders assess and discuss the extent to which they should link their strategy to the BRI’s narrative of globalization: is a long-term commitment to BRI warranted, or is the perception that the BRI is just a story mostly attractive on a case-by-case basis?

##### Proposition 4:

For *the BRI globalization narrative to go viral*, its matching institutions and rules must provide sustained perceived benefits to both Chinese and non-Chinese business interests and society.

### BRI counternarratives as barriers

The BRI as a globalization narrative that goes viral will impact other variables, first and foremost institutional change and the globalization institutions. The viral BRI narrative will also affect firm strategy and international firm performance levels. At the same time, the BRI is now in the realism space of geopolitics, where the success or failure of narratives with international business relevance is decided in part on the relative power of nation states.

An adaptation of Mearsheimer’s realism theory of international relations (Mearsheimer 2018) would hold that the smooth accommodation of a rising power’s narrative by an incumbent is but a “liberal dream.” Interestingly, the structuralist nature of realism theories and the Thucydides trap (Allison 2017) imply that even if the BRI espoused the cardinal values of Western globalization narratives, its fate would still be that of the antagonist. Hagel (2011) explains that some narratives unify and others divide. Both globalization and deglobalization narratives are viewed as divisive because they create discrete winners and losers in international business. In other circumstances, if political economy coalitions in the West, such as in the finance, e-commerce, or energy sectors, expected tangible benefits from the BRI, they would align with, participate in, and contribute to the narrative. However, the present trade conflicts and deglobalization illustrate the special situation in which the BRI operates. Global governance is being transformed by new and contending strategic narratives (Ellyatt 2018). In that context, the United States is frontally challenging the BRI, as Lee and Lee explain (Lee and Lee 2020). However, an even more extreme step exists: to cast the BRI as a “weaponized narrative.”

“[A] weaponized narrative seeks to undermine an opponent’s civilization, identity […] by generating complexity, confusion, and political and social schisms. It can be used tactically as part of explicit military or geopolitical conflict; or strategically, as a way to reduce, neutralize, and defeat a civilization, state, or organization. Done well, it limits or even eliminates the need for armed force to achieve political and military aims.” (Allenby and Garreau 2017).

In fact, the BRI has already been conceived of as such a narrative: a recent piece by the Asia Society titled *Weaponizing the Belt and Road Initiative* poses the unambiguous question, “Is the BRI a vehicle for creating an expanded Chinese-dominated regional ecosystem that disadvantages the U.S. and likeminded states militarily as well as commercially?” (Russel and Berger 2020). Counternarratives are barriers to BRI influence. However, can the BRI really be perceived as a “weaponized narrative”? More generally, is China using narratives as weapons as Bay (2018) claims? The pandemic is upping the ante by creating yet another area for narrative competition (Schifrin 2020). The outcome of the BRI narrative versus counter-BRI narratives contest will influence the rules of international business and impact business advantages.

In a context not distorted by non-business forces, competitive value-creating firms in both China and the West support narratives and institutions favoring globalization. They fight off narrative challenges from losers of globalization, whose inefficient models cause them to seek protection and rents that work to narrativize political, geopolitical, or technological facts for institutional change toward deglobalization. Instead, in the tense climate of 2020, rent-seekers everywhere appear to trump value creators and align themselves with narratives of deglobalization (see Table 1). The Thucydides trap—a narrative itself despite its structuralist claims—is now a force in the market for deglobalization narratives. The virality of deglobalization narratives and the reinforcing dynamic between institutions and narratives is captured in the following statement: “With each round of cascading tariffs, Trump has bullied more American companies into becoming protectionist” (Bown 2020). The BRI counternarratives and the institutional barriers to the BRI often have their foundations in domestic political economy considerations.

## Implications

### Doubling down on the BRI

Academics concerned about institutional change leading to net economic gains deeply worry about headlines such as “Coronavirus: China and U.S. in ‘new Cold War’ as relations hit lowest point in ‘more than 40 years’ spurred on by pandemic” (Bermingham and Zhou 2020). Any step toward the fragmentation of the international business arena is a loss. Of course, the likelihood of a direct conflict between the two contenders in this dialectic can be close to nil in the nuclear era, but a proxy might be in the cards (Witt 2019: 691). Such a conflict would play out primarily in the market for narratives; however, as we have seen, narratives can determine opportunity sets and constraints for international firms. The extreme nature of some of the narratives being suggested (e.g., compensation for COVID-19, Yeung 2020) might presage the worst-case scenario of regionalization that splits the globe into competing regions (Moyo 2019; Witt 2019). Hopefully, counter-BRI deglobalization narratives will not incorporate populist sentiments and add fuel to anti-China or to anti-Western narratives. However, because the once unthinkable is no longer impossible, this study must turn prescriptive. First, we examine what BRI ought to be for its stakeholders.

1. The BRI and the world economy: To matter, the BRI needs to deliver a context with a better general economic outlook. In this regard, Shiller (2019: 3) notes, “Just as a disease, an economic narrative, a story that is connected to things that matter in the economy, rises like an epidemic to influence a large fraction of the population.”
2. The BRI and far-reaching rules: A narrative will perform best on the back of ambitious rules. The US-led post-WWII globalization arrangements and supranational institutions had welcomed an “Open China” policy when China initiated economic reform since late 1970s. Trade rules offering unimpeded and consistent access to possibly the world’s largest market are a decisive narrative asset for globalization. China would be the center of the BRI, but the BRI’s narrative would make the definitive shift from “China internationalization” to “globalization,” which would give China’s narrative a pull not unlike that once enjoyed by the Washington Consensus.
3. The BRI and foreign winners: Ideas certainly can play a transformative role in society (Polanyi-Levitt 2012: 5), but globalization ideas should be formulated as narratives that embody values and that are participative, open-ended, and benefit broad international political economy coalitions. An “Open China” narrative would represent institutional change with far-reaching consequences. Such a narrative applied at the BRI space itself could materialize with specific and inclusive rules in emerging sectors, such as digitalization and health care, or in third-party market cooperation arrangements around transparency and global best practices (Zhang 2019: 324). The BRI narrative and its derived rules must make competitive foreign interest groups winners across the BRI space.

### Making the BRI part of international business strategy

For international businesses in the challenging post-2020 global context, the prescriptive implications of this article include the following:

1. Develop narrative capital. Oliver (1997: 707) discusses institutional capital as a firm’s resource and strategies that support it as a *sine qua non* for a sustainable competitive advantage. However, international businesses must bet on the right narratives to develop institutional capital. How well firms align their own narratives with the BRI’s narrative (or with counter-BRI alternatives) will determine their performance. Narrative capital becomes essential to international business strategy.
2. Walk the “thin line.” Geopolitical monitoring is becoming essential as firms navigate their selections between contending narratives and develop their own narratives. The dilemma for foreign multinationals in Shanghai, for Hong Kong investors in the United States, or for participants in natural resource projects in Russia is that firms’ narratives must be acceptable to China, the United States, and other relevant countries. International firms must carefully tread a very thin line. The alternative is the persistence of regionally constrained international strategies (Rosa et al. 2020) and the continued failure of multinationals to grow out of their home regions and become genuinely international firms contributing to and benefiting from globalization.
3. Seek narrative leadership. Narratives are central to addressing many of today’s key leadership challenges (Denning 2006: 42). Developing a powerful firm narrative is an important challenge for senior managers (Buckup and Casas 2018). Proactively and creatively managing international firms’ narrative assets, whether aligned with the BRI or with deglobalization, should be a strategic priority for business leaders.

### Implications for scholarship on BRI

This article aims to elicit directions for future research such as:

1. Conjecture testing. All four suggested conjectures are falsifiable and meant to be tested, including, for instance, research designs to resolve the values embodied by the BRI narrative, for which stakeholders these values matter, and whether international firms’ behaviors toward the BRI narrative are opportunistic or reflect active leadership and participation.
2. Quantification of the BRI narrative. If the BRI is a narrative, how viral and how effective is it? Empirical work leveraging public data sources, policy papers, or social networks would seek to understand the degree to which and where the BRI elicits contagious interest. Likewise, the data ascertain in what directions the BRI narrative evolves in the minds (and hearts) of Chinese and non-Chinese stakeholders alike. In this regard, such an observatory would establish virality and related patterns and then determine the confluences of the BRI with relevant phenomena, such as de/globalization, foreign direct investments (FDI), or firm strategies.
3. Relationship between the BRI narrative and dependent variables. This study suggests that the nature and strength of the relationship between the BRI viral narrative of globalization as an independent variable and the selected dependent variables is a fundamental future research direction. The dependent variables would first include institutional changes in globalization. Concreate research questions would be numerous, such as whether BRI and RCEP are “complimentary in design” (Ji 2020) or whether, in contrast, RCEP makes the BRI globalization narrative less relevant. Firm-level research questions would look beyond perceived economic performance (Proposition 4) and center on the actual variance in international firm performance associated with aligning firm strategies and narratives with the BRI narrative for globalization in both the Chinese market and BRI countries.

Academics, executives, and entrepreneurs all view the rollout of BRI institutions, the reconfiguration of global value chains, technology disruptions, the integration of Eurasia, trade wars, and regionalization with trepidation and concern. Will the BRI narrative bring about meaningful institutional change and increased opportunities for improving firm performance? Its success, virality and impact will depend on its pull with Chinese domestic political and economic coalitions and with international businesses and broader social interests. The four propositions provide a framework for forecasting how that will go. The framework incorporates the insights gained by addressing the first research question on the factors that drive institutional change, as well as the second question on the implications for international business practice and scholarship of the BRI narrative. More generally, however, this article nudges the decision-maker occupied with international strategy towards a deep and inclusive understanding of narratives in order to position the organization for global performance. This article has aimed to supply novel ideas and a stimulating framework for management scholars, decision makers, and leaders in the policy and corporate worlds.
